# Identification of Introgressed Alleles Conferring High Fiber Quality Derived From *Gossypium barbadense* L. in Secondary Mapping Populations of *G. hirsutum* L.

**DOI:** 10.3389/fpls.2018.01023

**Published:** 2018-07-18

**Authors:** Yu Chen, Guodong Liu, Hehuan Ma, Zhangqiang Song, Chuanyun Zhang, Jingxia Zhang, Junhao Zhang, Furong Wang, Jun Zhang

**Affiliations:** ^1^Key Laboratory of Cotton Breeding and Cultivation in Huang-Huai-Hai Plain, Ministry of Agriculture, Cotton Research Center of Shandong Academy of Agricultural Sciences, Jinan, China; ^2^College of Life Sciences, Shandong Normal University, Jinan, China; ^3^College of Agriculture, Nanjing Agricultural University, Nanjing, China

**Keywords:** fiber quality, introgression alleles, secondary mapping populations, quantitative trait loci (QTL), QTL cluster

## Abstract

The improvement of fiber quality is an essential goal in cotton breeding. In our previous studies, several quantitative trait loci (QTLs) contributing to improved fiber quality were identified in different introgressed chromosomal regions from Sea Island cotton (*Gossypium barbadense* L.) in a primary introgression population (Pop. A) of upland cotton (*G. hirsutum* L.). In the present study, to finely map introgressed major QTLs and accurately dissect the genetic contribution of the target introgressed chromosomal segments, we backcrossed two selected recombinant inbred lines (RILs) that presented desirable high fiber quality with their high lint-yielding recurrent parent to ultimately develop two secondary mapping populations (Pop. B and Pop. C). Totals of 20 and 27 QTLs for fiber quality were detected in Pop. B and Pop. C, respectively, including four and five for fiber length, four and eight for fiber micronaire, two and four for fiber uniformity, five and four for fiber elongation, and six and four for fiber strength, respectively. Two QTLs for lint percentage were detected only in Pop. C. In addition, seven stable QTLs were identified, including two for both fiber length and fiber strength and three for fiber elongation. Five QTL clusters for fiber quality were identified in the introgressed chromosomal regions, and negative effects of these chromosomal regions on lint percentage (a major lint yield parameter) were not observed. Candidate genes with a QTL-cluster associated with fiber strength and fiber length in the introgressed region of Chr.7 were further identified. The results may be helpful for revealing the genetic basis of superior fiber quality contributed by introgressed alleles from *G. barbadense*. Possible strategies involving marker-assisted selection (MAS) for simultaneously improving upland cotton fiber quality and lint yield in breeding programs was also discussed.

## Introduction

Cotton (*Gossypium* spp.) is the leading worldwide textile fiber crop and provides the majority of the raw materials for the textile industry. As both the textile industry rapidly develops and the demand for textile products continues to diversify, the high requirements for fiber quality continue to increase; therefore, the improvement of cotton fiber quality is very important (Kohel et al., 2001). The cotton genus (*Gossypium*) comprises 45 diploid (2n = 26) and six tetraploid (2n = 52) species (Grover et al., 2015). Among the four cultivated *Gossypium* species worldwide, the American allotetraploid species (*G. hirsutum* L. and *G. barbadense* L.) dominate worldwide cotton production and have nearly replaced the Old World diploid cultivars (*G. arboreum* L. and *G. herbaceum* L.). Owing to its high lint yield and broad adaptability, the upland cotton allotetraploid species, *G. hirsutum* L. (AD)_1_, is the most widely cultivated cotton species and is of primary economic importance in modern worldwide cotton production (Mehboob-ur-Rahman et al., 2012; Zhang et al., 2015).

The genetic diversity of cultivated upland cotton is limited (May et al., 1995; Mehboob-ur-Rahman et al., 2012; Tyagi et al., 2014). Natural variation among wild and cultivated relatives of crops is an under-exploited resource in plant breeding. The introgression of such genetic components or genes into the background of elite crop lines provides plant breeders an important opportunity to improve the agricultural performance of modern crop varieties. Conventional breeding efforts via interspecific hybridization have resulted in the transfer of specific genes and, consequently, useful traits such as favorable fiber quality and biotic or abiotic tolerances and so on into the upland cotton gene pool, such as *G. harknessii* (Meyer, 1973), *G. thurberi* (Culp and Harrell, 1973; Culp et al., 1979), *G. stockii* (Nazeer et al., 2014), *G. longicalyx* (Nacoulima et al., 2012) and *G. arboreum* (Ahmad et al., 2011; Chandra and Sreenivasan, 2011; Iqbal et al., 2015). In particular, germplasm with improved fiber quality conferred by chromosomal introgressions from other species within the genus *Gossypium* has been developed; one of those species is another cultivated allotetraploid cotton, Sea Island cotton (*G*. *barbadense* L.), whose lint yield is much lower than that of *G. hirsutum* L. but whose fiber quality is markedly superior. The developed germplasms have been used in breeding to improve the fiber quality of upland cotton (Mergeai, 2003; Zhou et al., 2003; Smith et al., 2008; Cao et al., 2014). However, due to the negative genetic correlation between fiber quality and lint yield (Zhou et al., 2003; Shen et al., 2007; Shang et al., 2015), the use of introgressed germplasm to improve fiber quality and lint yield simultaneously in upland cotton breeding is difficult (Meredith, 2005; Wang et al., 2011, 2013).

Mapping quantitative trait loci (QTLs) for fiber quality and yield have facilitated molecular marker-assisted selection (MAS) to simultaneously improve cotton yield and fiber quality (Duran et al., 2009); as such, the identification of stable QTLs is necessary to use MAS to improve cotton fiber quality. More than 1000 QTLs involved in cotton fiber quality have been identified (Said et al., 2013; Yu et al., 2014). However, most research is based on a mapping population developed by the interspecific hybridization of *G. hirsutum* × *G. barbadense* (Jiang et al., 1998; Zhang et al., 2002; Lacape et al., 2003, 2009; Nguyen et al., 2004; Guo et al., 2007; Yu et al., 2007; Zhao et al., 2012); therefore, the use of these QTLs for MAS in upland cotton breeding is extremely limited (Liang et al., 2013). Some introgressed alleles for fiber quality improvement from wild cotton species such as *G. tomentosum* (Zhang et al., 2011) and *G. klotzschianum* (Xu et al., 2012) in upland cotton have recently been identified. However, most of the studies on QTL mapping of fibre quality in which an intraspecific population of *G*. *hirsutum* introgressed with chromosomal segments from other species of *Gossypium* were used (Shen et al., 2007; Zhang et al., 2009, 2012; Sun et al., 2012; Li et al., 2016; Ma et al., 2017; Tan et al., 2018) did not pay close attention to the contributions of the introgressed genetic components (or alleles). Genetic dissection of the introgressed alleles for fiber quality improvement from other *Gossypium* species, especially from cultivated allotetraploid species with superior fiber quality, i.e., *G. barbadense* L, would be beneficial to MAS in upland cotton breeding with respect to the simultaneous improvement of fiber quality and lint yield.

In our previous study, using an introgression germplasm with superior fiber quality, we identified components introgressed from *G. barbadense* into upland cotton at a genome-wide level, and found that the majority of favorable alleles for fiber quality traits were derived from the introgression genomic components (Wang et al., 2011). By using F_2_ and F_2:3_ mapping populations, we further detected QTLs related to fiber quality and lint percentage and found that most of the favorable alleles were located on the introgressed chromosomal segments on Chr. 2, Chr. 3, Chr. 7, Chr. 16, Chr. 19, Chr. 23, and Chr. 25. In addition, major QTLs (*qFS-C7-1, qFL-C16-1, qFL-C25-1*, etc.) were subsequently detected (Wang et al., 2013). We had also constructed an RIL population for further searching stable QTLs for fiber quality improvement by MAS (Wang et al., 2016).

The objectives of this study were as follows: (1) Develop secondary introgression mapping population for anchoring the target introgressed chromosomal regions by backcrossing two selected RILs (Wang et al., 2016) that present desirable higher fiber quality with their high lint-yielding recurrent parent; (2) Accurately dissect the genetic contribution of the target introgressed chromosomal regions to improved fiber quality and consequently, investigate the genetic basis of the formation of excellent fiber quality traits in upland cotton; and (3) Finely map the introgressed major or stable fiber quality QTLs, and further evaluate the degree and direction (positive or negative) of the effects of those QTLs on the main lint yield traits, such as lint percentage. In addition, by using introgressed germplasm with superior fiber quality, we aimed to explore molecular breeding strategies for the simultaneous improvement of yield and fiber quality in upland cotton.

## Materials and Methods

### Plant Materials

Lumianyan22 (LMY22) was an upland Bt-cotton cultivar with high yield (high lint percentage) and medium staple developed by our research group. Luyuan 343 (LY343) was an upland cotton germplasm of high-fiber quality introgressed with chromosomal segments from *G. barbadense* cv. Ashimouni (Su et al., 2000; Wang et al., 2011). A recombinant inbred lines population was constructed using a cross of LMY22 × LY343 (Wang et al., 2016). We selected two high-fiber quality lines, R472 and R497, which include the target introgression chromosomal region (Supplementary Table S1), from the RIL population; and crossed with the recurrent parent, LMY22, for constructing mapping populations.

### Construction of the Secondary Mapping Populations, Phenotypic Valuation and Identification of Putative Introgression Genomic Components

The cross of R472 × LMY22 and R497 × LMY22 were performed at Experimental Station of Shandong Cotton Research Center (ESSCRC), Linqing County, Shandong province, China, in 2011. And the F_1_ plants were self-pollinated at Hainan Island in winter season of the year. The F_2_ populations comprising 514 (for Pop. B) and 544 (for Pop. C) individuals, were grown at ESSCRC and in routine cotton trial field in summer season of the year 2012. We named the two populations as Pop. B and Pop. C to be distinguished from the primary introgression populations (defined as “Pop. A” hereinafter) constructed by LY343 × LMY22. All F_2_ individuals were self-pollinated and the seed cotton samples were harvested. The seeds of each F_2_ individuals were homogenized and divided randomly into two similar populations of F_2:3_ family lines. The two secondary populations were planted separately at different environments in summer season of the year 2013. One was planted at Changsha (28°12′ N, 112°59′E), Hunan province of China, and the other was planted at ESSCRC (36°81′N, 115.71°13′E), Shandong province of China. The fiber quality and agronomical traits evaluations were performed on every individual (line) of all the three environments, and three datasets (E1: 2012, ESSCRC, F_2_; E2: 2013, ESSCRC, F_2:3_; E3: 2013, Hunan, F_2:3_) for the followed QTL mapping.

All fiber quality parameters of fiber length (FL, mm), fiber strength (FS, cN/Tex), fiber length uniformity (FU), fiber elongation (FE) and Micronaire value (FM, an integrated fiber quality parameter of fineness and maturity) were tested by the Supervision, Inspection and Test Center of Cotton Quality (SITCCQ), Ministry of Agriculture of China (MAC) using a High-Volume Precision Instrument (HVI; Zellweger-Uster, Knoxville, TN, United States). All other agronomical traits including lint percentage were evaluated by conventional methods of cotton breeding.

The introgression marker loci and putative introgression genomic components derived from *G. barbadense* L cv. Ashimouni were identified followed our previous report (Wang et al., 2011, 2013).

### Molecular Markers and Assays

Genomic DNA of all plant materials were extracted followed by the method reported by Paterson et al. (1993). The SSR (Simple Sequence Repeat) markers which have been exploited for QTL mapping in tetraploid cotton (Guo et al., 2007; Shen et al., 2007; Wang et al., 2008, 2013; Lacape et al., 2009; Zhang et al., 2009, 2011, 2012; Sun et al., 2012; Yu et al., 2014; Shi et al., 2015) were used in this study. All sequences of the SSR primers from CMD^1^ and CottonDB^1^ were synthesized by Sangon Biotech (Shanghai, China). The SSR markers which were polymorphic between R472 and LMY22, R497 and LMY22 were screened out as affirmative markers that used for mapping. The affirmative polymorphic primers were used to genotype F_2_ individual plants. The SSR assay were performed according to the procedure described by Zhang et al. (2002).

### Genetic Map Construction and QTL Analysis

All phenotypic data (including five parameters of fiber quality, and the main lint yield trait, i.e., lint percentage) were analyzed using SPSS17.0. The linkage map was constructed using Joinmap4.0 with a LODs core of 6.5. QTLs were detected by WinQTL Cartographer 2.5 using composite interval mapping (Wang et al., 2007). A stringent LOD threshold value were estimated by 1000 permutation test for all traits, and was used to declare the significant QTLs. The graphic representation of the linkage group and QTL marked were created by Map Chart 2.2 (Voorrips, 2002). QTL nomenclature was employed as before (Wang et al., 2011, 2013). QTLs detected in more than two data sets were considered as common or stable QTLs.

### Candidate Gene Analysis of the Stable QTL for Fiber Quality

The data of whole-genome sequences of *G. hirsutum* acc.TM-1 were downloaded from public databases^2^ (Zhang et al., 2015). The sequences between the adjacent markers for the stable QTL to genomic sequences were performed using SegHunter Software (Ye et al., 2010). Genes within the QTL mapped interval were defined as candidates responsible for the fiber quality trait. The candidate genes were identified by extracting the sequences for between the adjacent markers according to the position of the markers on corresponding chromosome, and comparing to the CDS sequences of the *G. hirsutum* acc.TM-1 to analyze the identity, alignment length and position. A bioinformatics analysis of annotated genes in the target regions was then performed.

### Quantitative RT-PCR

Total RNA was isolated from the developing cotton fibers [10-, 15-, 20-, and 25-day post anthesis (DPA)] of LMY22 and LY343 using a quick RNA Isolation Kit (Huayueyang, Beijing) according to the manufacturer’s protocol. The RNA qualities were detected by agarose gel electrophoresis, and RNA concentrations were measured using NanoPhotometer (ND2000). One μg RNA of each samples were prepared to perform cDNAs synthesis using PrimeScript RT Reagent Kit with gDNA Eraser (TaKaRa, Japan). In order to verify the temporal expression patterns of target genes in developing fibers, qRT-PCR was performed on the grounds of the protocol of SYBG^®^ Premix Ex Taq^TM^ II (TaKaRa, Japan) on the LightCycler^®^ 96 Real-Time PCR System (Roche, Switzerland). The constitutive expression gene *β*-Actin was used as internal reference to normalize the relative expression levels (forward primer: 5′-ATCCTCCGTCTTGACCTTG-3′, and reverse primer: 5′-TGTCCGTCAGGCAACTCAT-3′). Three biological and two technical replicates were performed to make the results credible. The relative gene expression level was calculated by the 2^-ΔΔC_t_^ method.

## Results

### Phenotypic Variation of Fiber Quality

To deeply dissect the genetic contribution of the introgressed *G. barbadense* alleles to improved fiber quality in *G. hirsutum*, we backcrossed two higher fiber quality lines to their recurrent parent to develop two secondary mapping populations. Compared with the female parent LMY22, the two parents from the RIL population, R472 and R497, presented significantly better fiber length, strength, and micronaire, but for fiber uniformity and fiber elongation, no significant difference was detected (Supplementary Table S2).

The phenotypic variation for fiber quality in the two populations is shown in Supplementary Table S3. The average fiber length, strength and micronaire in Pop. C were superior to those in Pop. B. The skewness and kurtosis values of the mapping populations showed that the fiber quality traits in three environments were normally distributed in Pop. B. In addition, the fiber quality traits in the three environments (except that the fiber uniformity in the environment of F_2_ LQ) were normally distributed in Pop. C.

### Construction of Genetic Map

**FIGURE 1:**
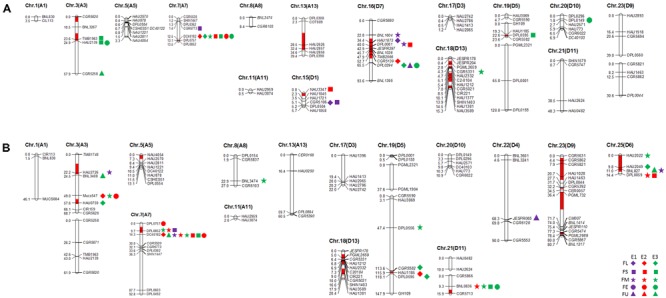
Quantitative trait loci (QTLs) for fiber quality detected in three environments by secondary mapping populations. **(A)**: QTLs for fiber quality detected in three environments by Pop. B. **(B)**: QTLs for fiber quality detected in three environments by Pop. C. The linkage group bars filled with red color indicated the putative introgression chromosomal regions or alleles. Deviated loci were shown with italics in linkage groups. The rhombus, square, pentagram, circular, and triangle in the right of the corresponding linkage groups represented for fiber length (FL), fiber strength (FS), micronaire (FM), fiber elongation (FE) and fiber uniformity (FU). The different color of purple, red and green represented the QTL detected in E1, E2, and E3, respectively.

In this study, 101 and 112 SSR markers (screened from 366 SSR primer pairs that were polymorphic in our primary mapping population; Wang et al., 2013) revealed polymorphism between the two selected RIL parents (R472 and R497) and the recurrent parent (LMY22). Herein, for Pop. B (LMY22 × R472), ninety-two marker loci were mapped to eighteen linkage groups, which were assigned to fifteen chromosomes, and nine loci were not linked to any group. The genetic map spanned 516.2 cM and accounted for 11.6% of the total allotetraploid cotton genome; the average distance between adjacent markers was approximately 5.61 cM. The minimum distance was 0.1 cM (Supplementary Table S4 and **Figure 1**). For Pop. C (LMY22 × R497), 105 marker loci were mapped to nineteen linkage groups on fifteen chromosomes, and seven loci were not linked to any group. The genetic map spanned 822.4 cM and accounted for 18.48% of the total cotton genome; the average distance was approximately 7.83 cM between adjacent markers. The minimum distance was 0.1 cM (Supplementary Table S4 and **Figure 1**).

### Putative Introgressed Genomic Components

A total of 21 out of 92 mapped SSR loci were identified as putative introgressed *G. barbadense* loci in Pop. B. The introgressed loci were distributed on 11 introgressed segments assigned to seven chromosomes, i.e., Chr. 3, Chr. 7, Chr. 13, Chr. 15, Chr. 16, Chr. 18, and Chr. 19. The introgressions accounted for approximately 21.50, 1.48, 46.04, 19.83, 32.59, 41.965, and 7.57% of each chromosome, respectively (Supplementary Table S5). In addition, twenty of one hundred and five mapped SSR loci were identified as putative introgressed loci in Pop. C. These introgressed loci were distributed on 12 introgressed segments assigned to eight chromosomes, i.e., Chr. 3, Chr. 5, Chr. 7, Chr. 18, Chr. 19, Chr. 21, Chr. 23, and Chr. 25. The introgressions accounted for approximately 13.47, 27.91, 7.72, 15.62, 1.21, 12.69, 52.32, and 88.64% of each chromosome, respectively (Supplementary Table S6).

### QTLs for Fiber Quality Traits and Lint Percentage

A total of 43 QTLs for fiber quality and lint percentage traits were detected on 13 chromosomal regions; these QTLs explained 2.69–18.8% of the total phenotypic variation. Sixteen and twenty-three QTLs were detected in Pop. B and in Pop. C, respectively. Four of the QTLs were common to both Pop. B and C, and 12 QTLs were defined as stable QTLs (**Table 1** and **Figure 1**). The favorable alleles of 25 QTLs (60.98%) were derived from the secondary introgression parents (R472 or R497), whose superior fiber quality properties were derived from our primary introgression parent, LY343.

**Table 1:** The QTLs for fiber-related traits detected in F_2_ and F_2:3_ of the secondary introgression mapping population.

QTL	Data sets	Nearest marker	Chr.	Position (cM)^a^	LOD	*A*	*D*	*R*^2^(%)	Direction
**Fiber length**									
*qFL-C3-1*	C: F_2:3_ LQ	MUCS547	3	41.5	3.44	–0.26	–0.23	3.71	R497
	C: F_2:3_ HuN	HAU0759	3	62.6	3.92	–0.28	0.14	3.43	R497
*qFL-C7-1*	B: F_2:3_ LQ	DC40182	7	10.4	4.27	–0.25	0.25	4.00	R472
	B: F_2:3_ HuN	DC40182	7	12.4	7.17	–0.35	0.17	6.98	R472
	C: F_2:3_ LQ	DC40182	7	12.7	6.45	–0.35	0.13	5.38	R497
*qFL-C15-1*	B: F_2_ LQ	CGR5106	15	5.1	5.63	–0.4	–0.21	4.73	R472
*qFL-C16-1*	B: F_2_ LQ	HAU1873	16	36.4	4.70	0.43	0.05	4.23	LMY22
*qFL-C16-2*	B: F_2:3_ LQ	CGR5139	16	50.9	6.41	0.36	0.26	6.11	LMY22
	B: F_2:3_ HuN	DPL0294	16	59.7	4.86	0.31	–0.1	5.03	LMY22
*qFL-C19-1*	C: F_2:3_ LQ	HAU1185	19	116.9	6.62	–0.37	–0.15	5.54	R497
	C: F_2:3_ HuN	DPL0595	19	121.1	4.82	–0.32	–0.17	4.31	R497
*qFL-C19-2*	C: F_2:3_ HuN	CGR5582	19	91.4	5.24	–0.48	–0.28	10.77	R497
*qFL-C25-1*	C: F_2:3_ HuN	HAU2049	25	8	4.58	–0.31	–0.09	3.95	R497
**Fiber uniformity**									
*qFU-C3-1*	B: F_2:3_ HN	CGR5258	3	46.9	3.51	–0.33	0.13	5.11	R472
*qFU-C3-2*	C: F_2:3_ HuN	BNL3408	3	27.5	4.21	–0.26	0.01	3.90	R497
*qFU-C7-1*	C: F_2:3_ HuN	DC40182	7	14.3	3.52	–0.22	0.05	2.91	R497
*qFU-C16-1*	B: F_2_ LQ	DPL0294	16	78.5	3.14	–0.42	0.40	4.46	R472
*qFU-C22-1*	C: F_2_ LQ	JESPR065	22	40.6	4.13	1.46	1.83	3.93	LMY22
*qFU-C25-1*	C: F_2:3_ HuN	BNL827	25	11	6.89	–0.27	–0.25	5.68	R497
**Micronaire**									
*qFM-C3-1*	C: F_2_ LQ	HAU2726	3	22.2	3.56	0.08	0.16	2.84	LMY22
*qFM-C3-2*	C: F_2:3_ HuN	MUCS547	3	42.5	5.6	0.11	0.00	5.13	LMY22
*qFM-C7-1*	B: F_2:3_ HuN	DC40182	7	8.4	18.41	0.25	0.06	18.80	LMY22
	C: F_2_ LQ	DC40182	7	12.7	14.41	0.3	–0.05	12.80	LMY22
	C: F_2:3_ LQ	DC40182	7	21.3	11.41	0.19	0.00	10.61	LMY22
	C: F_2:3_ HuN	DC40182	7	20.3	12.25	0.17	0.03	11.51	LMY22
*qFM-C7-2*	C: F_2:3_ HuN	DPL0852	7	9.7	15.91	0.17	0.01	12.27	LMY22
	C: F_2:3_ LQ	DPL0852	7	9.7	12.18	0.18	–0.03	9.64	LMY22
*qFM-C8-1*	C: F_2:3_ HuN	BNL3474	8	14.9	3.91	–0.09	0.05	3.87	R497
*qFM-C16-1*	B: F_2_ LQ	BNL1604	16	22.8	3.18	–0.05	0.16	2.69	R472
*qFM-C16-2*	B: F_2_ LQ	DPL0061	16	41	5.34	–0.21	0.03	4.94	R472
*qFM-C18-1*	B: F_2:3_ HuN	CGR5331	18	4.3	5.79	0.15	0.02	4.37	LMY22
*qFM-C19-1*	C: F_2:3_ HuN	DPL0556	19	72.4	4.22	0.14	0.03	8.75	LMY22
*qFM-C21-1*	C: F_2:3_ LQ	BNL0836	21	9.3	3.80	–0.1	–0.01	2.91	R497
	C: F_2:3_ HuN	BNL0836	21	9.3	4.30	–0.07	–0.06	3.13	R497
*qFM-C25-1*	C: F_2_ LQ	BNL827	25	11	6.57	0.21	–0.02	5.43	LMY22
	C: F_2:3_ HuN	HAU2022	25	0	4.27	0.09	0.03	3.01	LMY22
	C: F_2:3_ LQ	DPL0059	25	13	10.53	0.17	0.01	8.52	LMY22
**Fiber strength**									
*qFS-C3-1*	B: F_2:3_ HuN	TMB1963	3	23.6	3.80	–0.89	0.13	3.47	R472
	B: F_2:3_ HuN	HAU2139	3	35.9	3.68	–1.03	0.44	5.21	R472
*qFS-C7-1*	B: F_2:3_ LQ	DC40182	7	10.4	17.21	–1.12	0.19	15.05	R472
	B: F_2:3_ HuN	DC40182	7	12.4	5.69	–0.95	0.63	5.57	R472
	C: F_2:3_ LQ	DC40182	7	20.3	19.64	–1.24	0.35	18.47	R497
	C: F_2:3_ HuN	DC40182	7	20.3	3.00	–0.72	0.01	3.00	R497
*qFS-C7-2*	B: F_2_ LQ	CGR6773	7	4.4	6.02	–0.54	0.33	5.66	R472
*qFS-C7-3*	C: F_2_ LQ	DPL0852	7	5	9.02	–0.63	0.53	9.18	R497
*qFS-C15-1*	B: F_2_ LQ	CGR5106	15	5.1	4.87	–0.47	–0.22	4.12	R472
	B: F_2:3_ LQ	NAU3347	15	1	4.76	–0.55	0.04	3.74	R472
*qFS-C16-1*	B: F_2:3_ LQ	DPL0061	16	40	4.66	0.59	0.16	3.52	LMY22
*qFS-C19-1*	B: F_2:3_ LQ	DPL0595	19	21.3	3.47	–0.41	–0.46	2.91	R472
*qFS-C21-1*	C: F_2:3_ HuN	BNL0836	21	13.3	5.13	–0.88	0.12	4.74	R497
*qFS-C25-1*	C: F_2:3_ LQ	DPL0059	25	13	8.55	–0.78	0.12	6.69	R497
**Fiber elongation**									
*qFE-C3-1*	B: F_2:3_ HuN	HAU2139	3	36.9	3.19	0.14	–0.04	4.11	LMY22
*qFE-C3-2*	C: F_2:3_ LQ	MUCS547	3	38.5	8.15	0.06	0.03	9.06	LMY22
*qFE-C7-1*	B: F_2:3_ LQ	DC40182	7	12.4	5.83	0.04	–0.03	5.15	LMY22
	C: F_2:3_ LQ	DC40182	7	16.3	8.06	0.05	–0.03	7.18	LMY22
*qFE-C7-2*	C: F_2:3_ LQ	DPL0757	7	4	6.69	0.05	–0.03	6.16	LMY22
*qFE-C16-1*	B: F_2:3_ HuN	DPL0294	16	74.5	3.49	–0.16	–0.09	6.04	R472
*qFE-C20-1*	B: F_2:3_ HuN	DPL0149	20	1	3.25	–0.11	0.11	3.27	R472
*qFE-C21-1*	C: F_2:3_ LQ	BNL0836	21	9.3	5.09	0.04	–0.02	4.21	LMY22
**Lint percentage**									
*qLP-C13-1*	C: F_2:3_ LQ	DPL0894	13	47.4	6.69	0.01	0.00	8.87	LMY22
*qLP-C25-1*	C: F_2:3_ LQ	HAU2049	25	9.8	6.73	0.01	0.00	5.77	LMY22

*B represent a Pop. B which is constructed by R472 and LMY22; C represent a Pop. C which is constructed by R497and LMY22. ^*a*^The position of QTLs on linkage map, because of the difference in size and parents of mapping groups, resulting the different position on different linkage groups.*

#### Fiber Length

Eight QTLs explaining 3.43–10.77% of the phenotypic variation were detected. Three and four QTLs were detected in Pop. B and Pop. C, respectively. One QTL was found in both populations. Four QTLs (*qFL-C3-1, qFL-C7-1, qFL-C16-2*, and *qFL-C19-1*) were identified as being stable, of which *qFL-C7-1* was detected in three data sets. This QTL explained 4.00–6.98% of the phenotypic variation and was tightly linked to marker DC40182 (**Table 1** and **Figure 1**), while the other QTLs, *qFL-C3-1, qFL-C16-2*, and *qFL-C19-1*, were detected in two data sets.

#### Fiber Uniformity

Six QTLs for fiber uniformity were detected and explaining 2.91–5.68% of the phenotypic variation. All the QTLs were detected in only one data set. The favorable alleles of the QTLs were derived from superior-fiber quality parents except that of *qFU-C22-1* (**Table 1** and **Figure 1**).

#### Fiber Micronaire

Eleven QTLs were detected, and four (*qFM-C7-1, qFM-C7-2, qFM-C21-1*, and *qFM-C25-1*) of QTLs were identified as stable QTLs. The *qFM-C7-1* was detected in four data sets, explaining the phenotypic variation from 10.61 to 18.80% and tightly linked to marker DC40182. Regarding the other three stable QTLs, *qFM-C7-2* was detected in two data sets and explained 9.64–12.27% of the phenotypic variation, *qFM-C21-1* was detected in two data sets and explained 2.91–3.13% of the phenotypic variation, and *qFM-C25-1* was detected in three data sets and explained 3.01–8.52% of the phenotypic variation. With the exception of those of the QTL *qFM-C7-*2, the favorable alleles were derived from the high-quality secondary introgression parents. The other QTLs were detected in only one data set (**Table 1** and **Figure 1**).

#### Fiber Strength

A total of nine QTLs for fiber strength were detected in the two secondary introgression mapping populations. Five and three QTLs were detected in Pop. B and Pop. C, respectively. One QTL was common to both populations. Three of the QTLs (*qFS-C3-1, qFS-C7-1*, and *qFS-C15-1*) were identified as stable QTLs, and their favorable alleles were derived from the secondary introgression parents. The stable QTL *qFS-C7-1* was detected in four data sets and explained 3.00–18.47% of the phenotypic variation; this QTL was tightly linked to marker DC40182. The other stable QTLs, *qFS-C3-1* and *qFS-C15-1*, were detected in two data sets and explained 3.47–5.21% and 3.74–4.12% of the phenotypic variation, respectively. The remaining six QTLs were detected in only one data set, and with the exception of those of the QTL *qFS-C16-1*, the favorable alleles were derived from the superior-fiber quality parents (**Table 1** and **Figure 1**).

#### Fiber Elongation

A total of seven QTLs for fiber elongation were detected in the two mapping populations. These QTLs explained 3.27–9.06% of the phenotypic variation. One QTL, *qFE-C7-1*, was detected in two data sets and defined as a stable QTL; this QTL was also tightly linked to marker DC40182. The other six QTLs were detected in only one data set (**Table 1** and **Figure 1**).

#### Lint Percentage

Two QTLs for lint percentage were detected in Pop. C and explained 5.57–8.87% of the phenotypic variation. Both QTLs were detected in only one data set, and the favorable alleles were derived from the high lint-percentage parent LMY22 (**Table 1** and **Figure 1**).

### Common QTLs Across Multiple Populations

**FIGURE 2:**
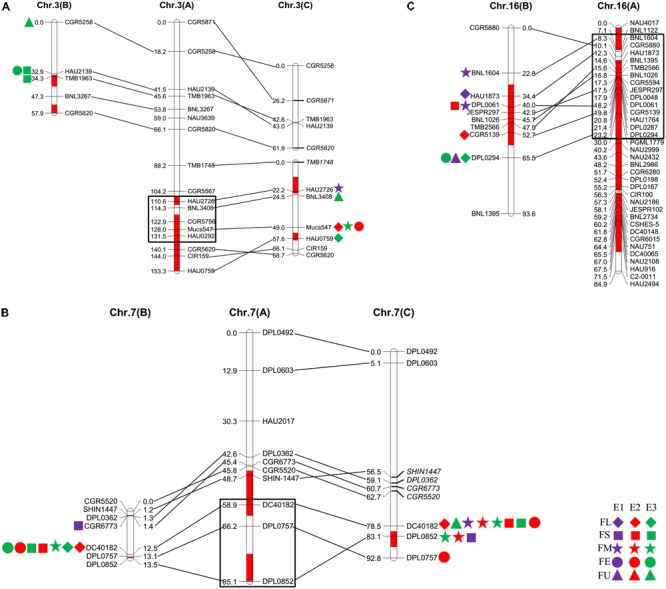
The common QTLs detected among different populations. **(A–C)** was representing the common QTLs on Chr. 3, Chr. 7, and Chr. 16, respectively. The rhombus, square, pentagram, circular, and triangle in the side of the corresponding linkage groups represented for fiber length, fiber strength, micronaire, fiber elongation, and fiber uniformity. The different color of purple, red, and green represented that the QTLs were detected in E1, E2, and E3, respectively. The black box represented for the region of the QTLs in Pop. A.

The QTLs for a defined trait detected in two different populations for which the confidence intervals overlap are considered “common” QTLs (Kenis et al., 2008). In the present study, we comprehensively compared the QTLs that were detected in the two secondary introgression populations (Pop. B and Pop. C) with the previously detected QTLs in the primary population (Pop. A) (Wang et al., 2011) and found that five common QTLs were shared between different populations, including two for fiber length, one for fiber strength, and two for fiber elongation (**Table 2** and **Figure 2**). The *qFE-C3-1* QTL for fiber elongation in the introgressed region of Chr. 3 was common to both Pop. A and Pop. C, and the *qFL-C16-1* QTL for fiber length on Chr. 16 was common to both Pop. A and Pop. B. In the introgressed region of Chr. 7, three common QTLs, including *qFL-C7-1, qFS-C7-1*, and *qFE-C7-1*, were common to Pop. A, Pop. B and Pop. C. These three QTLs were also detected in the RIL population developed from the same parents (Wang et al., 2016). These common QTLs are considered stable QTLs and can be applied via MAS as favorable introgressed alleles to improve fiber quality in cotton breeding.

**Table 2:** Common QTLs detected in three different populations.

Traits	Chr.	Pop. A				Pop. B				Pop. C			
		Name of QTLs	Markers nearest to QTLs	Environment	*R*^2^(%)	Name of QTLs	Markers nearest to QTLs	Environment	*R*^2^(%)	Name of QTLs	Markers nearest to QTLs	Environment	*R*^2^(%)
FL	7	*qFL-C7-1*	DC40182	F_2_ LQ,	9.9	*qFL-C7-1*	DC40182	F_2:3_ LQ	4	*qFL-C7-1*	DC40182	F_2:3_ LQ	5.38
			DC40182	F_2:3_ LQ	9.41		DC40182	F_2:3_ HuN	6.98	–	–	–	–
	16	*qFL-C16-1*	CGR5139	F_2:3_ HaiN	9.04	*qFL-C16-2*	CGR5139	F_2:3_ LQ	6.11	–	–	–	–
FS	7	*qFS-C7-1*	DC40182	F_2_ LQ	26.21	*qFS-C7-1*	DC40182	F_2:3_ LQ	15.05	*qFS-C7-1*	DC40182	F_2:3_ LQ	18.47
			DC40182	F_2:3_ HaiN	13.74		DC40182	F_2:3_ HuN	5.57		DC40182	F_2:3_ HuN	3
		*qFS-C7-2*	DPL0852	F_2_ LQ	27.09	–	–	–	–	*qFS-C7-3*	DPL0852	F_2_ LQ	9.18
FE	3	*qFE-C3-3*	MUCS547	F_2:3_ LQ	12.31	–	–	–	–	*qFE-C3-2*	MUCS547	F_2:3_ LQ	9.06
	7	–	–	–	–	*qFE-C7-1*	DC40182	F_2:3_ LQ	5.15	*qFE-C7-1*	DC40182	F_2:3_ LQ	7.18
		–	–	–	–		DC40182	F_2:3_ HuN	2.28	–	–	–	–
		*qFE-C7-1*	DPL0757	F_2_ LQ	14.73	–	–	–	–	*qFE-C7-2*	DPL0757	F_2:3_ LQ	6.16

*Pop. A represented the population developed from a cross of LMY22 × LY343, which was constructed as a primary mapping population in our previous study; Pop. B and Pop. C represented the population from the cross of R472 × LMY22 and R497 × LMY22, respectively; FL, fiber length; FS, fiber strength; FE, fiber elongation; LQ, LinQing; HaiN, HaiNan; HuN, HuNan; “-” represented no QTL was detected in this population.*

### QTL Clusters Associated With Fiber-Related Traits

A QTL cluster is defined as a chromosomal region that contains multiple QTLs for more than three associated traits. In this study, we identified five QTL clusters which comprised of 27 QTLs and were distributed on Chr. 3, Chr. 7, Chr. 16, and Chr. 25 (**Table 3**). Chromosome 3 contained two QTL clusters, both of which harbored three QTLs and were detected in Pop. B and Pop. C. The stable QTL *qFE-C3-2* detected in Pop. C and Pop. A resided in the region of c3-cluster-2 (**Table 3** and **Figure 2**). An important QTL cluster, the c7-cluster, was located on Chr. 7 and was detected in Pop. B and Pop. C. This QTL cluster was also detected in almost all data sets of Pop. A and of the RIL population developed from the same parents. Two major QTLs, *qFS-C7-1* and *qFM-C7-1*, which explained more than 10% of the phenotypic variation for fiber strength and micronaire, were co-located in this cluster region, suggesting that key genes controlling fiber development may reside in this region. This QTL cluster region was flanked by markers DPL0757 and DC40182, and the approximate physical distance was 185 kb (**Table 3** and **Figure 2**). The QTL cluster c16-cluster, contained five fiber quality QTLs, and a stable QTL, *qFL-C16-1*, resided within this cluster region (**Table 3** and **Figure 2**). The c25-cluster detected in Pop. C contained four QTLs, and the approximate physical distance was 209.4 kb (**Table 3**).

**Table 3:** Distribution of QTL clusters in Pop. B and Pop. C.

Cluster name	Adjacent marker	Traits included	Approximate position on chromosome (cM)	Number of QTLs	Name of QTLs in population
c3-cluster-1	TMB1963-CGR5268	FS + FE + FU	23.6–57.9	3	*qFS-C3-1*-Pop. B
					*qFU-C3-1*-Pop. B
					*qFE-C3-1*-Pop. B
c3-cluster-2	MUCS547-HAU0759	FL + FM + FE	49.0–57.6	3	*qFL-C3-1*-Pop. C
					*qFM-C3-2*-Pop. C
					*qFE-C3-2*-Pop. C
c7-cluster	DPL0757-DC40182	FL + FS + FM + FE + FU	12.5-13.1	12	*qFL-C7-1*-Pop. B
					*qFS-C7-1*-Pop. B
					*qFM-C7-1*-Pop. B
					*qFE-C7-1*-Pop. B
					*qFL-C7-1*-Pop. C
					*qFS-C7-1*-Pop. C
					*qFS-C7-3*-Pop. C
					*qFM-C7-1*-Pop. C
					*qFM-C7-2*-Pop. C
					*qFU-C7-1*-Pop. C
					*qFE-C7-1*-Pop. C
					*qFE-C7-2*-Pop. C
c16-cluster	DPL0061-DPL0294	FL + FS + FM + FE + FU	40.0-65.5	5	*qFL-C16-2*-Pop. B
					*qFS-C16-1*-Pop. B
					*qFM-C16-2*-Pop. B
					*qFU-C16-1*-Pop. B
					*qFE-C16-1*-Pop. B
c25-cluster	HAU2049-DPL0059	FL + FS + FM + FU	9.8–14.4	4	*qFL-C25-1*-Pop. C
					*qFS-C25-1*-Pop. C
					*qFU-C25-1*-Pop. C
					*qFM-C25-1*-Pop. C

*Pop. B: the population of R472 × LMY22, Pop. C: the population of R497 × LMY22; FL, Fiber length; FS, Fiber strength; FM, Fiber micronaire; FU, Fiber uniformity; FE, Fiber elongation.*

### Prediction and Comparative Analysis of Candidate Genes in the c7-Cluster Region

**FIGURE 3:**
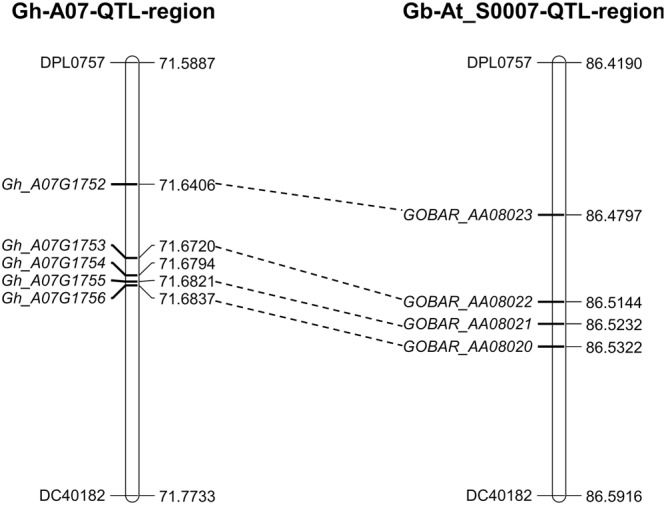
Annotated genes within the 185-kb in c7-cluster region between the *G. hirsutum* and *G. barbadense*. *Gh_A07G1752* (RING/U-box superfamily protein, RHA2B); *Gh_A07G1753* (CONSTANS-like 9, COL9); *Gh_A07G1755* (Plant protein of unknown function, DUF868); *Gh_A07G1754* and *Gh_A07G1756* (without any function). *GOBAR_AA08020* (uncharacterized membrane protein); *GOBAR_AA08021* (conserved hypothetical protein); *GOBAR_AA08022* (B-box type zinc finger protein with CCT domain isoform 1); *GOBAR_AA08023* (RING/U-box superfamily protein, putative).

The 0.6 cM region between DPL0757 and DC40182 corresponds to a 185-kb genomic region on chromosome A07 of the *G. hirsutum* acc. TM-1 genome. According to the reference genome annotation of *G. hirsutum* (Zhang et al., 2015), the 185-kb genomic region contains five predicted genes: those coding for the RING/U-box superfamily protein (RHA2B, *Gh_A07G1752*), CONSTANS-like 9 (COL9, *Gh_A07G1753*), and a plant protein of unknown function (DUF868) (*Gh_A07G1755*), as well as other two genes with no known function (*Gh_A07G1754* and *Gh_A07G1756*) (Supplementary Table S7). Coding sequence comparisons between the chromosomes of *G. hirsutum* and *G. barbadense* revealed some asymmetry, and the four genes in the *G. hirsutum* genome have homoeologous counterparts in the *G. barbadense* reference genome sequence, i.e., *Gh_A07G1752* is homoeologous to *GOBAR_AA08023, Gh_A07G1753* is homeologous to *GOBAR_AA08022, Gh_A07G1755* is homeologous to *GOBAR_AA08021*, and *Gh_A07G1756* is homeologous to *GOBAR_AA08020* (**Figure 3**).

**FIGURE 4:**
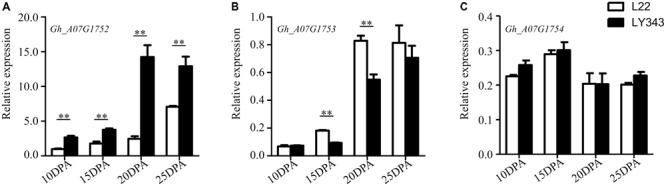
RT-qPCR expression assay of genes in c7-cluster region during fiber development. **(A)** RING/U-box superfamily protein (RHA2B, *Gh_A07G1752*), **(B)** CONSTANS-like 9 (COL9, *Gh_A07G1753*), and **(C)** Not any function (*Gh_A07G1754*). The white bars are L22, and the black bars are LY343. Error bars indicate the standard deviation of three biological replicates. ^∗∗^Denote significantly differential expression at *p* < 0.01.

The expression levels of the five annotated genes within this QTL region were tested by RT-qPCR using fiber tissue from four developmental stages (10, 15, 20, and 25 DPA) in LMY22 and LY343. Via the sequence alignment of *G. hirsutum* acc. TM-1 (Zhang et al., 2015), single locus-specific primers of the five candidate genes were designed for the divergent coding region (Supplementary Table S8). The relative expression levels in both parents (LMY22 and LY343) are shown in **Figure 4**. The expression level of *Gh_A07G1752* were gradually increased from 10 DPA to 25 DPA, especially in LY343; compared with those in LMY22, the expression levels of *Gh_A07G1752* in LY343 exhibited an approximately six-fold increase at the 20 DPA stage. The gene *Gh_A07G1753* was significantly differentially expressed between LMY22 and LY343, especially at 15 and 20 DPA. The expression levels of *Gh_A07G1754* did not significantly differ between LMY22 and LY343 during the four developmental stages. Moreover, we did not detect any expression for *Gh_A07G1755* or *Gh_A07G1756* during the four developmental stages.

## Discussion

### Genetic Effects of Stable Introgressed QTLs and QTL Clusters Detected in Secondary Mapping Populations

To identify introgressed exogenous chromosomal regions, we identified 158 out of 322 (158/322) SSR marker loci in a genetic map constructed using a primary introgression mapping population developed from a cross of LMY22 × LY343; these markers represented putative introgressed *G. barbadense* alleles (Wang et al., 2013). The 322 polymorphic SSR markers were screened from a total of 18,467 SSRs previously exploited in interspecific mapping (Nguyen et al., 2004; Guo et al., 2007; Lacape et al., 2009; Zhang et al., 2009, 2011). The total introgressions covered 646.91 cM, which accounted for approximately 12.86% of the tetraploid cotton genome, and were located on 41 introgressed chromosomal segments assigned to 20 chromosomes (Wang et al., 2013). QTL mapping demonstrated that most of the favorable alleles for fiber quality QTLs were located in the introgressed chromosomal regions derived from the primary introgression parent, i.e., LY343. Stable QTLs and/or QTL clusters were identified in only a few of the introgressed chromosomal regions, including the introgressed regions on Chr. 7 and Chr. 25. These introgressed chromosomal regions might contain important genes for superior fiber quality (Wang et al., 2013, 2016).

In the present study, by backcrossing secondary introgression upland cotton lines that present both desirable higher fiber quality and relatively better lint yield performance with their high lint-yielding recurrent parent, we developed a secondary introgression mapping population to anchor the target introgressed chromosomal regions. The mapping parents were individual lines from the RIL population developed from the primary introgression parent (Wang et al., 2016). The secondary introgression parent for developing Pop. B, i.e., R472, contains the introgressed regions on Chr. 7, Chr. 13, Chr. 15, Chr. 16, Chr. 18, Chr. 19, and Chr. 20, while the parent for developing Pop. C, i.e., R497, contains the introgressed regions on Chr. 3, Chr. 5, Chr. 7, Chr. 8, Chr. 18, Chr. 19, Chr. 21, Chr. 22, Chr. 23, and Chr. 25 (Supplementary Table S1). These parents contain the key introgressed chromosomal regions that have favorable alleles for fiber quality QTLs or QTL clusters previously detected by us (Wang et al., 2011, 2013, 2016).

Using these two secondary introgression mapping populations (Pop. B and Pop. C), we detected 41 QTLs for fiber quality, and these QTLs were distributed on all the above mentioned chromosomal regions (**Table 1**). As expected, most of the QTLs for fiber quality were mapped to the introgressed chromosomal regions, and most of the favorable alleles of these QTLs were contributed by the introgressed genomic components (**Figure 1**) We detected introgressed fiber quality QTLs or QTL clusters on Chr. 3, Chr. 7, Chr. 15, Chr. 16, Chr. 18, and Chr. 19 in Pop. A, and we detected introgressed QTLs or QTL clusters on Chr. 3, Chr. 7, Chr. 19, Chr. 21, and Chr. 25 in Pop. B. In contrast with the mapping results for Pop. A previously reported by us (Wang et al., 2011, 2013), the present results showed five groupings of common QTLs in the introgressed region of Chr. 7, i.e., *qFL-C7-1*-Pop. A, *qFL-C7-1*-Pop. B and *qFL-C7-1*-Pop. C; *qFS-C7-1*-Pop. A, *qFS-C7-1*-Pop. B and *qFS-C7-1*-Pop. C; *qFS-C7-2*-Pop. A and *qFS-C7-3*-Pop. C; *qFE-C7-1*-Pop. A, *qFE-C7-1*-Pop. B and *qFE-C7-1*-Pop. C; and *qFE-C7-1*-Pop. A and *qFE-C7-2*-Pop. C, among which the three QTLs for fiber length, strength, and elongation were detected in all the mapping populations (**Figure 2**). As we reported recently, these three QTLs were also detected in RIL populations in multiple environments (Wang et al., 2016) and were reasonably stable introgressed QTL clusters for fiber quality. In addition, mapping via linkage analysis (Zhang et al., 2009; Sun et al., 2012; Xu et al., 2012; Cao et al., 2015; Shi et al., 2015; Jamshed et al., 2016; Fang X. et al., 2017) and genome-wide association studies (GWAS) (Islam et al., 2016; Su et al., 2016; Fang L. et al., 2017; Sun et al., 2017; Tan et al., 2018) have both been used to detect the major QTLs for fiber quality in this chromosomal region.

We detected one common QTL on Chr. 3, namely, *qFE-C3-1*-Pop. A and *qFE-C3-2*-Pop. C, for fiber elongation (**Figure 2**). We also detected one pair of common QTLs on Chr. 16, namely, *qFL-C16-1*-Pop. A and *qFL-C16-1*-Pop. B, for fiber length (**Figure 2**). Together with the QTL pairs detected on Chr. 7, without exception, those common QTLs were located in the introgressed chromosomal regions. In addition to the major or stable QTLs and QTL clusters for fiber quality located in the introgressed region of Chr. 25 (**Figure 1**), these introgression chromosomal regions might contain favorable alleles (or genes) for the desirable superior fiber quality. Detailed and systematic studies of these regions (or alleles) would be helpful for understanding the genetic and/or molecular mechanisms controlling the formation of superior fiber quality in the introgression germplasm.

### Candidate Genes for Superior Fiber Quality in the Putative Introgressed Chromosomal Region of Chr. 7

The fiber quality of LY343 was much better than those of LMY22 (Wang et al., 2011). We selected two high fiber-quality secondary introgression lines, i.e., R472 and R497, which contained overlapping introgressed fragments on some chromosomes (Chr. 7, Chr. 18, and Chr. 19), from the RIL population for backcrossing with LMY22 to construct secondary introgression mapping populations designated Pop. B and Pop. C. Using the secondary mapping populations, we mapped the major and stable fiber quality QTLs in the putative introgressed region of Chr. 7 within a 0.5 cM interval between markers DPL0757 and DC40182. According to the reference genome of *G. hirsutum* L. acc. TM-1 (Zhang et al., 2015), we found that five genes are present in the stable QTL region between DPL0757 and DC40182, namely, *Gh_A07G1752, Gh_A07G1753, Gh_A07G1754, Gh_A07G1755*, and *Gh_A07G1756* (Supplementary Table S7). *Gh_A07G1752*, a small but important gene, encodes a RING/U-box superfamily protein that plays regulatory roles both in the development of a variety of organisms and in the dramatic increase in cellular abscisic acid (ABA) levels and resulting drought tolerance (Ko et al., 2006; Xia et al., 2013). In the present study, the expression levels of *Gh_A07G1752* gradually increased in the four fiber developmental stages (**Figure 4**). *Gh_A07G1753* encodes the zinc finger protein CONSTANS-LIKE 9 (COL9), a member of the CONSTANS-LIKE gene family, and by repressing the expression of CO, COL9 is involved in the regulation of flowering time, concomitantly reducing the expression of FT and delaying floral transition (Cheng and Wang, 2005). Furthermore, based on the Gene Ontology results, *Gh_A07G1753* was recently predicted to encode a transcription factor involved in cotton fiber development (Islam et al., 2016). *Gh_A07G1755*, which encodes a plant protein of unknown function (DUF868), may be a new a gene that needs to be analyzed in the future. *Gh_A07G1754* and *Gh_A07G1756* have no known function according to the annotation for *G. hirsutum* L. acc. TM-1 (Zhang et al., 2015). Cotton fiber development is a complex process, and fiber development include four overlapping stages: fiber initiation, cell elongation, secondary wall deposition, and maturation (Lee et al., 2007). Many transcription factors which were involved in metabolic pathways such as phytohormone signaling, energy metabolism of cell, fatty acid metabolism, secondary metabolism and other signaling pathways are related in this process (Hande et al., 2017). So more detailed research is needed to identify the introgressed gene(s) that is (are) involved in cotton fiber development and contribute to superior fibre quality for this study.

### QTL Clusters and QTL Hotspots for Fiber Quality and Their Relationships With Introgressed Genomic Components

The existence of QTL clusters has been reported in cotton (Qin et al., 2008; Jiang et al., 2009; Said et al., 2013, 2015; Li et al., 2016; Zhang et al., 2016; Si et al., 2017) and other organisms (Xie et al., 2008; Mallard et al., 2013). Analyses of the reported 1,223 QTLs for yield and fiber quality, disease resistance and other traits across the *Gossypium* genome revealedthe presence of QTL clusters and/or specific trait QTL hotspots on almost all of the tetraploid chromosomes except Chr.9, Chr.13, Chr.20, and Chr.22 (Said et al., 2013). This finding indicates that genes related to specific traits are more concentrated within certain regions of the genome than others.

In our study, several QTLs that influence fiber quality were detected in the same chromosomal regions, indicating that QTL clusters and hotspots were also present. We detected five clusters: c3-cluster-1, c3-cluster-2, c7-cluster, c16-cluster, and c25-cluster. As mentioned above, the c7-cluster contained five annotated or predicted genes (Supplementary Table S7). There are many reports about QTL clusters of fiber quality on Chr. 7 (Zhang et al., 2009; Sun et al., 2012; Xu et al., 2012; Cao et al., 2015; Shi et al., 2015). However, no reports mentioned QTL clusters within the DPL0757-DC40182 interval. In the DPL0757-DC40182 interval located on Chr. 7 in Pop. B and Pop. C, *qFL-C7-1, qFS-C7-1, qFM-C7-1* and *qFE-C7-1* influenced fiber quality, including fiber length, strength, micronaire and elongation. This finding indicated that the DPL0757-DC40182 interval located on Chr. 7 is a QTL cluster and a hotspot interval for fiber quality. In addition to our previously reported mapping results in Pop. A (Wang et al., 2011, 2013), the QTL *qFS-C7-1* was detected in seven data sets (F_2:3_ in LQ and HuN in both Pop. B and Pop. C; F_2_ in LQ, F_2:3_ in LQ and HaiN in Pop. A), and except in one data set, was tightly linked to marker DC40182 (Pop. A and the F_2:3_ generation in LQ) (Wang et al., 2013). This QTL explained 3–26.21% of the phenotypic variation. As such, the QTL *qFS-C7-1* is a stable major QTL. It provides an underlying basis for cloning QTLs for both understanding the genetic mechanism of the superior fiber formation and deep mining the introgressed alleles (genes) associated with fiber development to further design and precisely breed (via MAS) for improved fiber quality in upland cotton.

### Evaluation of the Genetic Effects of the Introgressed Fiber Quality QTLs and/or QTL Cluster on Lint Yield Traits and Possible MAS-Based Strategies for Simultaneously Improving Fiber Quality and Lint Yield in Cotton Breeding

Cotton lint yield is mainly determined by the number of bolls in per area (NB), boll weight (BW) and lint percentage (or gin turnout), among these lint percentage is an important factor for determining cotton fiber production. Several studies have shown that fiber quality has a negatively genetically correlated with lint yield (Scholl and Miller, 1976; Zhou et al., 2003; Shen et al., 2007; Yu et al., 2013). Therefore, this phenomenon is a major obstacle with respect to the simultaneous improvement of lint yield (mainly lint percentage) and fiber quality in upland cotton breeding programs (Wang et al., 2013, 2016).

A total of 41 QTLs for fiber quality and two QTLs for lint percentage were detected in this study; most of those QTLs for fiber quality were related to the introgressed chromosomal regions or introgressed alleles derived from *G. barbadense*, and the favorable alleles of 25 QTLs are introgressions (**Table 1** and **Figure 1**). Interestingly, the favorable alleles of fourteen of seventeen (14/17) fiber length- and strength-related QTLs were introgressions. Only the favorable alleles of three QTLs on Chr. 16 originate from LMY22, i.e., they were not introgressions. This result further indicated that the introgressed alleles derived from *G. barbadense* play a key role in the formation of superior fiber quality, especially fiber length and strength.

Two QTLs for lint percentage (*qLP-C13-1* and *qLP-C25-1*) were identified in one of the secondary mapping populations (Pop. C). The favorable alleles of both QTLs are derived from LMY22. One of the QTLs, *qLP-C25-1* was clustered with *qFL-C25-1*, and they are both tightly linked to marker HAU2049 (**Table 1**). The favorable alleles of *qLP-C25-1* and *qFL-C25-1* originate from LMY22 and the secondary introgression parent, respectively. This finding indicated that these introgressed alleles are favorable for improving fiber length but it is not desirable for improving lint percentage. Therefore, regardless of whether the negative effect is caused by linkage drag or pleiotropism, this QTL and its tightly linked marker are not suitable for the simultaneous improvement of fiber quality and lint yield in cotton breeding.

We previously detected five QTLs for lint percentage (Wang et al., 2013); these QTLs were consistent with the lint percentage QTLs reported by Shi et al. (2015). Recently, using a RIL population in multiple environments, we also detected nine QTLs for lint percentage (Wang et al., 2016). One of the QTLs, namely, *qLP16.1*, located on Chr. 16 (D07) was detected in four environments, and the favorable allele originated from the introgression parent; unfortunately, we detected negative correlations between lint percentage and fiber quality traits in this introgressed chromosomal region. Chr. 16 is homeologous to Chr. 7 (A07), which contain two QTL clusters for fiber quality (**Figure 1** and **Table 3**). This finding suggests that polyploid lineages may determine the variations in subgenome contributions to QTLs (Fonceka et al., 2012). However, we detect no negative effects on lint percentage from the fiber quality-related QTLs and/or QTL clusters located on the introgressed chromosomal region of Chr. 7. Therefore, we believe that these fiber quality QTLs and/or QTL clusters are favorable for MAS to improve fiber quality in cotton breeding.

The simultaneous improvement of fiber quality and lint yield by conventional breeding methods is slow and difficult (Zhang et al., 2012). Molecular markers constitute a foundation for constructing a high-density linkage map to exploit effective markers (new SSRs, SNPs, and InDels) (Wang et al., 2015) and lay a foundation both for dissecting additional QTLs for lint percentage, and fiber quality and for stacking favorable alleles for the fiber quality and lint percentage mapping in non-homologous regions, enhancing the possibility of using MAS to improve both fiber quality and lint yield (Wang et al., 2013). We detected seven stable QTLs for fiber quality in this study (**Table 3**), and most of the QTLs have no negative effects on lint percentage. Notably, the fiber quality QTL cluster in the introgressed chromosomal region of Chr. 7 had significant genetic effects on fiber strength, length, micronaire. In addition, the genetic distance was 0.6 cM between the adjacent molecular markers, which is inaccordance with requirements.

Importantly, the QTLs for lint percentage, whose favorable alleles originate from non-introgression upland cotton parent, were mainly detected on Chr. 5, Chr. 15, Chr. 17, Chr. 18, and Chr. 22 using the genetic populations involved in introgressions from *G. barbadense* in previous studies (Wang et al., 2011, 2013, 2016) and other reports (Jenkins et al., 2006; Yu et al., 2013; Shi et al., 2015). Notably, these QTLs for lint percentage were distributed on the chromosomes that are not harboring the major QTLs for superior fiber quality. In addition, we detected no mutual negative effects on fiber quality and lint percentage in the majority of the fiber quality and lint percentage QTLs and/or QTL clusters, which is extremely important. Accordingly, we concluded that favorable elite alleles or QTLs can be pyramided via MAS for the simultaneous improvement on lint yield and quality in upland cotton breeding.

## Author Contributions

JZ and FW designed the experiments. GL, YC, JhZ, CZ, FW, and JZ performed field trials, phenotypic evaluation, and data collection. YC and JxZ contributed to the preparation of cotton RNA samples. HM performed the analysis of QTL mapping data, with the help of FW for the analysis of raw data. ZS performed the qRT-PCR. YC and FW drafted the manuscript. JZ revised the manuscript. All authors read and approved the final manuscript.

## Conflict of Interest Statement

The authors declare that the research was conducted in the absence of any commercial or financial relationships that could be construed as a potential conflict of interest. The reviewer BZ declared a shared affiliation, though no other collaboration, with one of the authors JhZ to the handling Editor.

## Footnotes

1 https://www.cottongen.org/

2 http://mascotton.njau.edu.cn/
